# The primary care provider’s role in providing supportive and palliative care for patients in chronic renal failure

**DOI:** 10.4102/safp.v62i1.5238

**Published:** 2020-12-11

**Authors:** René Krause, Nicola Wearne, Tsepo Motsohi, Bianca Davidson

**Affiliations:** 1 School of Public Health and Family Medicine, Faculty of Health Sciences, University of Cape Town, Cape Town, South Africa; 2 Department of Internal Medicine, Faculty of Health Sciences, University of Cape Town, Cape Town, South Africa

**Keywords:** chronic kidney chronic, disease renal failure, end-stage kidney disease, palliative care, primary care

## Abstract

Primary care providers are at the core of providing supportive and palliative care to patients with chronic kidney disease in South Africa. Although dialysis is not always needed, and sometimes not appropriate, for all patients with end-stage kidney disease, there is always supportive and palliative care that can be provided to patients and families to improve outcomes. This article explores the referral pathways, renal preservation, supportive and palliative care and, finally, health system interventions that can improve comprehensive care. The integration of renal supportive and palliative care is a relatively new concept in the paradigm of care and will require advocacy and research to ensure all South African patients have access throughout the trajectory of illness.

## Introduction

Primary care providers are at the core of providing supportive and palliative care (PC) to patients with chronic kidney disease (CKD) in South Africa. Chronic kidney disease in South Africa is a growing concern in the primary care services because of the rising incidence of non-communicable diseases resulting in end-stage kidney disease (ESKD). Furthermore, dialysis is not always available and sometimes not appropriate for all patients with ESKD in the South African setting.^1^ Despite these challenges, the principle of Universal Health Coverage should be followed to ensure promotive, preventive, curative, rehabilitative services and PC for patients with CKD.^2^ The primary care clinics are well positioned to deliver supportive and PC to patients on an outpatient basis in line with Primary Health Care principles.^2^ More specifically, community health centres (CHC) have the multidisciplinary resources to buttress PC’s comprehensive team approach.^3^ Nephrologists and PC-trained providers are limited by numbers. This makes primary care providers central to provide supportive care and PC to patients and families. In 2019, a consensus statement was produced by nephrologists, family physicians, PC providers and healthcare managers on core interventions that may improve care for patients in the South African setting.^1^ This article will draw from the consensus document and primary care experiences to highlight the role of the primary care provider in delivering these services.

The prevention of ESKD is core to primary care. High-risk individuals include those with diabetes, hypertension, urological disorders, human immunodeficiency virus (HIV), a family history of kidney disease and those with drug addiction (e.g. methamphetamines and cocaine). When any of the indications as shown in Box 1 are identified, referral to a nephrologist is indicated. Patients should be considered to have CKD when their estimated glomerular filtration rate (eGFR) is less than 60 millilitres (mL)/min/1.73 suqare metrs (m^2^) for more than 3 months or if they have small kidneys on ultrasound (< 9 centimetres [cm]).^4^ Referral to the nephrologist should occur when renal function declines to an eGFR of < 30 mL/min/1.73 m^2^. However, referral to tertiary level care for CKD should only occur if the patient is a potential candidate for the dialysis and transplant programme. The following groups of patients are not eligible for chronic kidney replacement therapy in the public health sector (see Box 2^5^).

BOX 1 Identification of patients requiring urgent nephrology referral.

- Presence of persistent or coexistent haematuria on dipsticks.
- Heavy proteinuria – urinary protein/creatinine ratio > 0.3 g/mmol despite ACE inhibitor or angiotensin receptor blocker (ARB) with a controlled blood pressure (BP).
- Abnormal renal ultrasound.
- Unexplained or rapid deterioration in renal function.
- Sudden deterioration in renal function after ACE inhibitor or ARB.
- Resistant hypertension on appropriate medication and compliant. (BP >140/90 mm Hg despite the use of three full-dose antihypertensive drugs, including a diuretic at full dose [hydrochlorothiazide at 25 mg bd and indapamide at 2.5 mg]).
- Human immunodeficiency virus-positive patients are at high risk of renal dysfunction and should be considered if any of the criteria above develops.
- Urology referral is more appropriate when there is urinary obstruction, recurrent renal infection and history of renal calculi.

ACE, angiotensin-converting enzyme; bd, twice daily.

BOX 2 Patients who are not eligible for chronic renal replacement therapy in the South African public health sector.

To note the following groups of patients are *not eligible for chronic renal replacement therapy* in the public health sector:

- Diabetics > 50 years
- Non-diabetics > 60 years of age
- Human immunodeficiency virus-positive patients with CD4 < 200 copies/mL and unsuppressed viral load
- Significant non-adherence to treatment
- Illicit drug use
- Multiple or significant organ damage (COPD, heart failure, ischaemic heart disease and liver disease).

*Source:* Moosa MR. Guideline: Priority setting approach in the selection of patients in the public sector with end-stage kidney failure for renal replacement treatment in the Western Cape Province; 2013. [cited November 2020] Available: http://s3.documentcloud.org/documents/19489/moosa-prioritysetting-policy-final-feb-24-2010-

COPD, chronic obstructive pulmonary disease.

At this juncture of identifying an individual with ESKD who is not for renal replacement, preservation of kidney function, detailed communication and advance care planning, pain and symptom control, psychosocial care and impeccable end of life care become the cornerstones of care. Primary care utilisation in its totality must be geared towards care for these patients to ensure that comprehensive care is available for all patients and their families with CKD.

Although some patients are not candidates for chronic dialysis programmes, renal preservation must continue focussing on the following aspects:

- Lifestyle changes: stop smoking, weight loss (body mass index [BMI] < 30 kilograms (kg)/m^2^), encourage exercise, low-salt diet.
- Control blood pressure (BP) to target < 140/90 millimetre of mercury (mmHg). If BP > 160/100, start two first-line drugs.^6^
  ■ Control BP to target at each opportunity.
  ■ Furosemide (loop diuretic) 40 milligrams (mg) – 120 mg 12 hourly if eGFR < 45 mL/min/1.73 m^2^.
- Consider starting treatment with an angiotensin-converting enzyme (ACE) inhibitor or angiotensin receptor blocker, titrate enalapril to 10 mg 12 hourly when a patient is proteinuric (however, recheck serum creatinine and potassium after 1 week of initiation).
- If eGFR > 60 mL/min/1.73 m^2^ on initial presentation, then routine blood will need to be repeated, preferably within 2 weeks.
- Avoid non-steroidal anti-inflammatory drugs (NSAIDs) and other potential nephrotoxins (tenofovir and aminoglycosides).
- Monitor potassium and creatinine at each visit if eGFR < 60 mL/min/1.73 m^2^. Avoid metformin and glibenclamide if eGFR < 45 mL/min/1.73 m^2^, preferably use gliclazide or insulin.
- Consider starting a statin in patients with high cardiovascular risk.
- Start calcium carbonate (Titralac) 1–2 tablets 8 hourly with meals and vitamin D 50 000 U weekly if eGFR < 30 mL/min/1.73 m^2^.

Communication around the disease, education on the preservation of renal function, reasons why the patient is not for dialysis and finally advance care planning should be initiated at this point. These discussions should preferably include family members and carers using a positive non-judgemental approach to ensure compassionate and effective communication. All team members at all levels of care (primary, secondary and tertiary) should apply this approach when patients have been declined for dialysis to ensure the same information be provided to patients and families across the continuum of care. These sessions should be structured, drawing from the total pain framework and the rest of the World Health Organization (WHO) PC principles of palliative care.^7^ This implies the inclusion of family members and carers in the sessions. Advance care planning is a discussion held with patients on the preferences, values and wishes around future care. It is best done early in the disease process when patients can actively participate in these discussions. Lastly, these discussions must be documented and, if the patient wishes, should be shared with family members.^8^

The pain and symptom burden of patients with CKD is well documented but poorly managed across the continuum of care.^9^ Common symptoms experienced by patients with CKD include uremic pruritus, sleep disorders, restless legs syndrome, anorexia, nausea and vomiting, uremic gastritis, constipation, diarrhoea, depression and pain.^10^ The causes, incidence and management in the South African context are outlined in the Renal Palliative and Supportive Guideline in South Africa as a consensus statement, which also draws from the South African Essential Medicine List (EML).^1,11^ These guidelines provide specific guidance on the pharmacological and non-pharmacological management of the symptoms and pain. The trajectory of illness of patients with ESKD may have periods of exasperation, and pain and symptoms need to be continuously reviewed and medication, especially diuretics, needs to be adjusted. The use of analgesia in kidney failure is complex, with the limited variety of availability of analgesia on the primary care EML.^11^ Morphine is currently the only drug available in the primary care EML for severe pain, but its use is complex as the metabolites accumulate in CKD.^1^ If morphine is used, it must be initiated at a very low dose on an ‘as required basis’, with close monitoring of the effect. Morphine should be prescribed with antiemetics and laxatives. The management of pain is a universal human right and the consensus in Cape Town was to treat the pain and to advocate for the availability for a wider variety of evidence-based analgesic medication in the primary care EML.^1^

As a patient’s condition deteriorates, decisions around end of life care must preferably be taken whilst the patient can still participate in decision-making with family involvement. Pain and symptom control must become paramount and the management strategy should then change to focus on symptom control (fluid management and diuresis) and pain management (morphine). Importantly, pill burden must be limited by stopping non-beneficial treatment (e.g. calcium carbonate, vitamin D and simvastatin). Notably, it is the place of death that is best discussed in advance. Admission to hospital in a patient known with ESKD (not for dialysis) must be carried out with specific reason and false hopes of improvement must not be created with families. If the patient and family wish to receive home care, appropriate home care must be initiated in the form of home-based carer teams and hospice care, if available.

Access to comprehensive kidney care will not be possible if the primary care system is not used in its totality. Trained ward-based home-based carer teams are an essential foundation for a successful PC service. Following a community-oriented primary care (COPC) model, each team would be supported by a clinical nurse practitioner or professional nurse and medical officer with PC modular training, at a minimum, and access to more specialised kidney and palliative services if more complex situations arise.^12^ Laboratory services, community occupational therapy and physiotherapy are all required to ensure comprehensive care is provided to the patients.

Further health system adjustments that can assist in the improvement of care include embedded stationery prompts and supportive standards of practice that are aligned to the guidelines. These could assist clinicians with the early recognition of clients who qualify for PC interventions. For renal palliative care, these prompts would be most useful if aligned to the care of patients with hypertension, diabetes and HIV because patients suffering from these epidemic diseases comprise the majority of kidney failure clients who do not qualify for renal replacement therapy in the public service.^13^

There are numerous ways to measure the impact of PC interventions in the literature.^14^ Many of these can be used as clinical governance quality assurance tools to assist specialist family physicians to support CHC teams using quality improvement cycles. Some of these criteria include clinical care measures using stationery audits, family feedback surveys and staff development training statistics. Renal supportive and palliative care are new concepts in the South African PC context and further understanding of the patients’ needs and how they respond to care is important because of our varied cultures and limited resources. Although we are in the initial phases of unpacking the South African needs of ESKD patients, it is recommended that supportive care and palliative care for CKD patients should be embedded in the pathways of patients with clear communication between healthcare providers from tertiary, secondary and primary care and the same message to patients and families.

## Conclusion

The care of patients with CKD occurs commonly in primary care and the active integration of supportive and PC must be normalised within the paradigm of care. This can only be achieved by the following: firstly, further PC training for staff members at primary care; secondly, system adjustments to ensure supportive care and PC is integrated into all care pathways; and, finally, closer collaboration between the tertiary, secondary and primary care healthcare providers. Palliative care is not an excuse for forgoing dialysis nor for withholding further active care. It is active care until the very end and beyond.
